# Hypervirulent *emm*59 Clone in Invasive Group A *Streptococcus* Outbreak, Southwestern United States

**DOI:** 10.3201/eid2204.151582

**Published:** 2016-04

**Authors:** David M. Engelthaler, Michael Valentine, Jolene Bowers, Jennifer Pistole, Elizabeth M. Driebe, Joel Terriquez, Linus Nienstadt, Mark Carroll, Mare Schumacher, Mary Ellen Ormsby, Shane Brady, Eugene Livar, Del Yazzie, Victor Waddell, Marie Peoples, Kenneth Komatsu, Paul Keim

**Affiliations:** Translational Genomics Research Institute, Flagstaff, Arizona, USA (D. Engelthaler, M. Valentine, J. Bowers, E.M. Driebe, P. Keim);; Arizona Department of Health Services, Phoenix, Arizona, USA (J. Pistole, S. Brady, E. Livar, V. Waddell, K. Komatsu);; Northern Arizona Healthcare, Flagstaff (J. Terriquez, L. Nienstadt, M. Carroll);; Coconino County Public Health Services District, Flagstaff (M. Schumacher, M.E. Ormsby, M. Peoples);; Navajo Division of Health, Window Rock, Arizona, USA (D. Yazzie);; Northern Arizona University, Flagstaff (P. Keim)

**Keywords:** group A Streptococcus, streptococci, pyogenes, bacteria, GAS, emm59, genomic epidemiology, polytomy WGST, Native Americans, southwestern United States, Arizona, California, New Mexico, Minnesota, Oregon, Canada

## Abstract

The hyper-virulent *emm*59 genotype of invasive group A *Streptococcus* was identified in northern Arizona in 2015. Eighteen isolates belonging to a genomic cluster grouped most closely with recently identified isolates in New Mexico. The continued transmission of *emm*59 in the southwestern United States poses a public health concern.

Several cases of invasive group A *Streptococcus* (GAS) disease were detected in January 2015 in a northern Arizona hospital. A substantive percentage of the cases were associated with a homeless shelter and a local jail; outbreak case-patients were predominantly male and Native American. Other studies have shown an increase in infection risk for invasive GAS in Native American/First Nations populations (*1*,*2*), and outbreaks within this population in Arizona have been previously documented (*3*). Whole genome sequence analysis determined that the hypervirulent subtype *emm*59 was present among the first cases analyzed in early 2015. *emm*59 is known to have caused a nationwide outbreak of invasive GAS in Canada during 2006–2009 (*4**,**5*), and cases and outbreaks have been reported in the United States (*6*–*8*).

## The Study

We identified isolates for sequencing from 29 invasive GAS cases diagnosed in patients in a northern Arizona hospital during January–July 2015 and randomly selected an additional 99 GAS isolates from a repository of >2,000 Arizona GAS isolates collected during 2002–2006 (no isolates from patients in Arizona were available for 2007–2014). Four additional isolates from central Arizona identified in 2015 were included in the analysis (Technical Appendix Table). All isolates were grown on 5% sheep blood tryptic soy agar plates (Hardy Diagnostics, Santa Maria, CA), and incubated at 37**°**C with 5% CO_2_. DNA was extracted by using a DNeasy Blood and Tissue Kit (QIAGEN, Valencia, CA, USA) following manufacturer’s protocol. Genomic DNA libraries were prepared by using the Nextera XT library prep kit (Illumina, San Diego, CA) and sequenced with paired-end reads (250 bp) on an Illumina MiSeq instrument, as previously described (*9*). The finished genome of the *emm*59 Canadian clone MGAS15252 (GenBank accession no. CP003116) and high-quality publicly available sequence-read data from 44 US isolates, from NCBI short read archive (BioProject #PRJNA194066), were included in the subsequent phylogenetic analyses. The final core genome (all nucleotide loci found in all genomes) for single-nucleotide polymorphism (SNP) detection was 1,636,024 bp (98.6% of reference).

We used NASP SNP analysis pipeline (http://tgennorth.github.io/NASP/) for whole-genome SNP typing as previously described (*10*). SNP matrices were developed for both the whole species and the *emm*59-only analyses. We used MEGA version 5.2.2 software (*11*) to generate maximum parsimony phylogenetic trees. Regions of high SNP density were identified as possible regions of recombination and were further analyzed for impact on the consistency index. Genomes were assembled by using UGAP (https://github.com/jasonsahl/UGAP). GAS *emm* subtypes were assigned by using BLAST (http://blast.ncbi.nlm.nih.gov/Blast.cgi), querying the study genome assemblies against the Centers for Disease Control and Prevention’s (CDC) *emm* type-specific sequence database (http://www.cdc.gov/streplab/m-proteingene-typing.html). We resolved dual *emm*-type hits using CDC’s *emm* typing Sanger sequencing primers (http://www.cdc.gov/streplab/protocol-emm-type.html) as a BLAST query and noting hit locations.

We identified 18 of the 29 contemporary northern Arizona isolates as subtype *emm*59; the remaining isolates were composed of 6 additional *emm* types: *emm*1 (n = 2), *emm*5 (n = 2), *emm*58 (n = 1), *emm*81 (n = 2), *emm*83 (n = 1), *emm*89 (n = 2), and *emm*94 (n = 1). The 99 historical and 4 contemporary background Arizona isolates included 25 distinct *emm* types (Technical Appendix Table). No *emm*59 isolates were identified in this background set, and none had been previously reported in Arizona.

**Figure 1:**
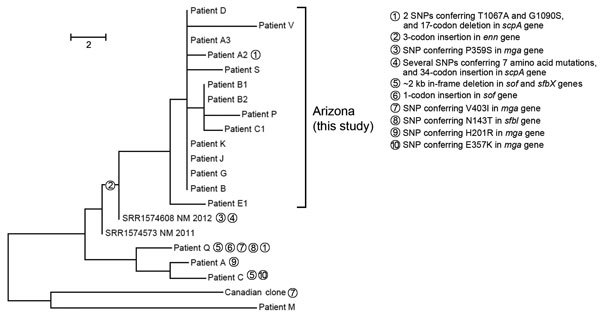
Phylogenetic single-nucleotide polymorphism (SNP) tree of *emm*59 isolates from a northern Arizona hospital displaying distribution of mutations in a 23kb positively selected region during invasive group A *Streptococcus* outbreak, southwestern United States. Maximum parsimony tree of all SNP loci (n = 58) in *emm*59 isolates (n = 18) from Arizona, 2 recent New Mexico isolate genomes, and the Canadian clone reference isolate MGAS15252. Consistency index = 1.0. Branch lengths represent numbers of SNPs between isolates; unit bar is in the figure. Numbered circles distinguish lineages of selected mutations in *scpA, enn, sfbl, mga, sfbx,* and *sof* genes in a 23-kb hotspot mutational region. Scale bar indicates SNPs.

The 18 Arizona *emm*59 cases occurred during January–July 2015 (Table). An *emm*59-only phylogenetic analysis demonstrated the apparent presence of multiple lineages of *emm*59 in the 2015 Arizona isolates (Figure 1). A distinct clone consisting of 14 of the 18 *emm*59 isolates were separated from each other by only 0–4 SNPs, genomically supporting the presence of an ongoing outbreak; >8 of these patients were epidemiologically linked to physical contact, cohabitation, or both with 1 other person (data not shown). The additional *emm*59 isolates make up additional lineages separated from one other by 8–28 SNPs. No recombination was identified among the Arizona isolates. A relatively large number of SNPs and indels were seen within an approximate 23-kilobase region (Figure 1). This region has been previously reported to contain mutational hotspots associated with virulence (*12*,*13*). Considering the presumptive positive selective force on this region, SNPs within the region were not included in the final phylogenetic analysis.

**Table:** Epidemiologic data for 18 case-patients with invasive *emm*59 group A *Streptococcus* infection, Arizona, USA, 2015*

Category	Value
Race	
American Indian or Alaskan Native	15 (83)
White	3 (7)
Sex	
F	4 (22)
M	14 (88)
Mean age, y (range)	40 (26–79)
Clinical information	
Cellulitis	7 (39)
Necrotizing fasciitis	5 (28)
Sepsis	9 (50)
Risk factors	
Injury	7 (39)
Alcohol abuse	10 (56)
Homeless	8 (44)
Living in shelter	5 (28)
Local jail term within ≈1 mo. of diagnosis	6 (33)

*Values are no. (%) patients except as indicated. Epidemiologic data based on available information.

**Figure 2:**
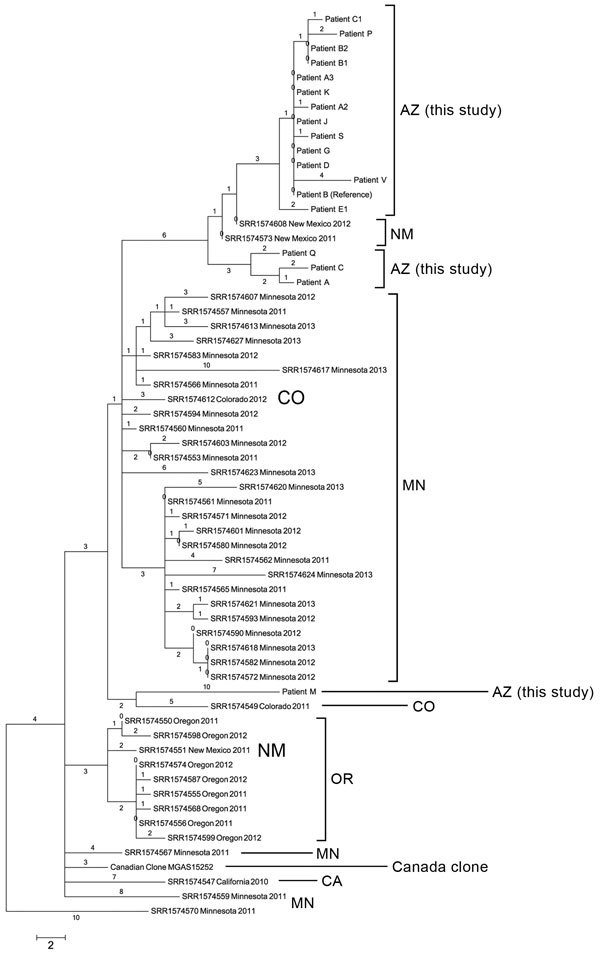
Phylogenetic single-nucleotide polymorphism (SNP) tree of *emm*59 isolates from Arizona during invasive group A *Streptococcus* outbreak in the southwestern United States, previously analyzed US *emm*59 isolates, and the Canadian clone. Maximum parsimony tree of all 177 SNP loci (44 parsimony informative SNPs) in *emm*59 isolates from Arizona (n = 18), Minnesota (n = 29), Oregon (n = 8), New Mexico (N = 3), Colorado (n = 2), and California (n = 1) and the Canadian clone reference isolate MGAS15252. Tree has regions of recombination removed and is rooted with Minnesota isolate SRR11574570. Consistency index = 1.0. Numbers above branches are SNP distances. Scale bar indicates SNPs.

When compared with all other publicly available US *emm*59 isolate genomes, nearly all the genomes identified in the United States were closely related to each other and to the Canadian clone MGAS15252; individual isolate SNP branch lengths ranged from 0 to 10 (Figure 2). The Arizona outbreak isolates were separated from 2 New Mexico isolates by 4 and 5 SNPs each; these isolates fell within the overall Arizona clade and were subsequently included in the Arizona-only phylogenetic analysis (Figure 1). Conversley, the isolate from patient M appears more distant from the larger Arizona population. The Arizona clades, with the exception of that of the isolate from patient M, all appear to arise from the large Minnesota polytomy. The previously estimated 1.3–2.1 SNPs/year mutation rates for GAS (*14*,*15*) further support the Arizona outbreak as being caused by a single clone, likely originating from New Mexico and being spread over 6–12 months.

## Conclusions

The *emm*59 subtype of GAS, the etiologic agent of a substantial nationwide outbreak of invasive GAS in Canada during 2006–2009 (*4*), is now present in Arizona, causing at least 1 outbreak of epidemiologically and genomically linked cases and several additional epidemiologically unrelated cases. The lack of *emm*59 in background isolates in Arizona from the previous decade, along with its low genetic diversity, suggests that *emm*59 emerged recently in Arizona. Following the *emm*59 epidemic in Canada, this subtype was subsequently seen in a few US states; a retrospective analyses of the Centers for Disease Control and Prevention Active Bacterial Core surveillance (ABCs) system (http://www.cdc.gov/abcs/reports-findings/survreports.pdf) identified 40 US *emm*59 isolates during 2000–2009 (*6*) and an additional 67 isolates during 2010–2012 (*7*). Of note, only 5 (of the 40 *emm*59 isolates from 2000–2009 (2 from Minnesota, 2 from California, and 1 from Oregon) appeared to be closely related to the Canadian clone (defined by the authors as being separated by <16 SNPs) (*6*); in contrast, all of the strains from the 2010–2012 survey appeared to be more closely related to the Canadian clone. The more recent ABCs analysis identified an increasing number of southwestern isolates, including 4 from Colorado and 6 from New Mexico (*7*), although no outbreaks were specifically described in these states (Arizona is not included in the ABCs system). Similar to this outbreak study, Olsen et al. (*7*), in an analysis of 60 MN *emm59* isolates from case-patients with identified race, determined that 25 (42%) were from Native Americans; of 5 isolates from New Mexico in that study, 3 were from Native Americans. 

Given the apparent distal nature of the Arizona/New Mexico isolates to the Minnesota population in our study, it is reasonable to propose an unidentified epidemiologic relationship between these case populations. However, caution must be used in drawing conclusions regarding the relationships of isolates from disparate geographic regions because only limited comparable sequence data from previous *emm*59 studies in the United States (*7*) were publicly available to compare to the Arizona isolates. Epidemiologic investigations, along with healthcare provider and patient education activities, are ongoing in Arizona to further determine the extent of the current outbreak and the associated risk factors and to help mitigate effects and limit or prevent further spread to at-risk populations.

Technical Appendix List of Arizona Group A Streptococcus strains.
