# Supplementary material for: Hypervirulent emm59 Clone in Invasive Group A Streptococcus Outbreak, Southwestern United States
Source: Emerg Infect Dis. 2016 Apr;22(4):734–8. doi: 10.3201/eid2204.151582 (PMC4806960; doi:10.3201/eid2204.151582)
Supplement: Technical Appendix — List of Arizona Group A Streptococcus strains. [file 15-1582-Techapp-s1.pdf]

Article DOI: http://dx.doi.org/10.3201/eid2204.151582

Hypervirulent *emm*59 Clone in Invasive Group A *Streptococcus* Outbreak, Southwestern United States

Technical Appendix

Technical Appendix Table. Arizona Group A Streptococcus strain list.

Sample name	Isolation year	EmmType
Patient V	2015	emm59
Patient T	2015	emm58
Patient S	2015	emm59
Patient R	2015	emm89
Patient Q	2015	emm59
Patient P	2015	emm59
Patient O	2015	emm94
Patient N	2015	emm81
Patient M	2015	emm59
Patient L	2015	emm81
Patient K	2015	emm59
Patient J	2015	emm59
Patient I	2015	emm89
Patient H	2015	emm1
Patient G	2015	emm59
Patient F	2015	emm5
Patient E1	2015	emm59
Patient E	2015	emm5
Patient D	2015	emm59
Patient C1	2015	emm59
Patient C	2015	emm59
Patient B2	2015	emm59
Patient B1	2015	emm59
Patient B	2015	emm59
Patient A3	2015	emm59
Patient A2	2015	emm59
Patient A1	2015	emm83
Patient A	2015	emm59
Patient 50	2015	emm1
15-AZDH-31896	2015	emm1
15-AZDH-31642	2015	emm89
15-AZDH-31316	2015	emm89
15-AZDH-31315	2015	emm89
06-AZDH-917	2006	emm1
06-AZDH-889	2006	emm1
06-AZDH-6998	2006	emm83
06-AZDH-6944	2006	emm1
06-AZDH-6629	2006	emm1
06-AZDH-653	2006	emm1
06-AZDH-6182	2006	emm156
06-AZDH-6118	2006	emm80
06-AZDH-5439	2006	emm81
06-AZDH-5161	2006	emm1
06-AZDH-4827	2006	emm1
06-AZDH-4311	2006	emm1
06-AZDH-3955	2006	emm28
06-AZDH-390	2006	emm22
06-AZDH-3232	2006	emm75
06-AZDH-3204	2006	emm1
06-AZDH-3102	2006	emm1
06-AZDH-310013	2006	emm75

Sample name	Isolation year	EmmType
06-AZDH-299002	2006	emm1
06-AZDH-2950	2006	emm3
06-AZDH-2909	2006	emm118
06-AZDH-286011	2006	emm28
06-AZDH-284001	2006	emm28
06-AZDH-283016	2006	emm76
06-AZDH-281	2006	emm1
06-AZDH-2550	2006	emm12
06-AZDH-2484	2006	emm41
06-AZDH-1564	2006	emm118
06-AZDH-1120	2006	emm83
05-AZDH-9295	2005	emm33
05-AZDH-8840	2005	emm1
05-AZDH-8819	2005	emm12
05-AZDH-7607	2005	emm118
05-AZDH-7138	2005	emm22
05-AZDH-649	2005	emm83
05-AZDH-5954	2005	emm78
05-AZDH-5712	2005	emm22
05-AZDH-5225	2005	emm12
05-AZDH-4797	2005	emm1
05-AZDH-4700	2005	emm87
05-AZDH-3549	2005	emm94
05-AZDH-341	2005	emm6
05-AZDH-3409	2005	emm76
05-AZDH-3375	2005	emm12
05-AZDH-11701	2005	emm80
05-AZDH-1139	2005	emm12
05-AZDH-11329	2005	emm92
05-AZDH-11117	2005	emm81
05-AZDH-10477	2005	emm12
04-AZDH-8764	2004	emm83
04-AZDH-8624	2004	emm1
04-AZDH-8516	2004	emm6
04-AZDH-7857	2004	emm1
04-AZDH-7507	2004	emm89
04-AZDH-7482	2004	emm28
04-AZDH-7102	2004	emm75
04-AZDH-6995	2004	emm12
04-AZDH-6727	2004	emm156
04-AZDH-6516	2004	emm1
04-AZDH-6188	2004	emm28
04-AZDH-6011	2004	emm114
04-AZDH-5802	2004	emm58
04-AZDH-5600	2004	emm76
04-AZDH-5454	2004	emm80
04-AZDH-4661	2004	emm6
04-AZDH-4596	2004	emm28
04-AZDH-4517	2004	emm2
04-AZDH-3925	2004	emm1
03-AZDH-924	2003	emm2
03-AZDH-739	2003	emm83
03-AZDH-693	2003	emm12
03-AZDH-546	2003	emm3
03-AZDH-376	2003	emm5
03-AZDH-3258	2003	emm87
03-AZDH-3091	2003	emm1
03-AZDH-296	2003	emm1
03-AZDH-2839	2003	emm1
03-AZDH-2776	2003	emm1
03-AZDH-2617	2003	emm75
03-AZDH-2592	2003	emm3
03-AZDH-2592	2003	emm3
03-AZDH-2525	2003	emm89
03-AZDH-2235	2003	emm3
03-AZDH-1872	2003	emm114
03-AZDH-1822	2003	emm3
03-AZDH-1666	2003	emm2
03-AZDH-1622	2003	emm89

Sample name	Isolation year	EmmType
03-AZDH-2454	2003	emm1
03-AZDH-1105	2003	emm48
02-AZDH-273	2002	emm22
02-AZDH-268	2002	emm1
02-AZDH-249	2002	emm12
02-AZDH-238	2002	emm75
02-AZDH-221	2002	emm156
02-AZDH-198	2002	emm12
02-AZDH-181	2002	emm156
02-AZDH-172	2002	emm75
02-AZDH-166	2002	emm41
02-AZDH-158	2002	emm1
02-AZDH-154	2002	emm1
